# Intelligent Epileptic Seizure Detection and Classification Model Using Optimal Deep Canonical Sparse Autoencoder

**DOI:** 10.3390/biology11081220

**Published:** 2022-08-15

**Authors:** Anwer Mustafa Hilal, Amani Abdulrahman Albraikan, Sami Dhahbi, Mohamed K. Nour, Abdullah Mohamed, Abdelwahed Motwakel, Abu Sarwar Zamani, Mohammed Rizwanullah

**Affiliations:** 1 Department of Computer and Self Development, Preparatory Year Deanship, Prince Sattam Bin Abdulaziz University, AlKharj 16278, Saudi Arabia; 2 Department of Computer Sciences, College of Computer and Information Sciences, Princess Nourah bint Abdulrahman University, P.O. Box 84428, Riyadh 11671, Saudi Arabia; 3 Department of Computer Science, College of Science & Art at Mahayil, King Khalid University, Abha 62529, Saudi Arabia; 4 Department of Computer Sciences, College of Computing and Information System, Umm Al-Qura University, Makkah 24382, Saudi Arabia; 5 Research Centre, Future University in Egypt, New Cairo 11745, Egypt

**Keywords:** deep learning, feature selection, EEG signals, epileptic seizure recognition, classification, krill herd algorithm

## Abstract

**Simple Summary:**

Epileptic seizures are a chronic and persistent neurological illness that mainly affects the human brain. Since the traditional way of diagnosing epileptic seizures is laborious and time-consuming, automated tools using machine learning (ML) and deep learning (DL) models may be useful. This paper presents an intelligent deep canonical sparse autoencoder-based epileptic seizure detection and classification (DCSAE-ESDC) model using EEG signals. The proposed DCSAE-ESDC technique involves two major processes, namely, feature selection and classification. The DCSAE-ESDC technique designs a novel coyote optimization algorithm (COA)-based feature selection technique for the optimal selection of feature subsets. Moreover, the DCSAE-based classifier is derived for the detection and classification of different kinds of epileptic seizures. Finally, the parameter tuning of the DSCAE model takes place via the krill herd algorithm (KHA).

**Abstract:**

Epileptic seizures are a chronic and persistent neurological illness that mainly affects the human brain. Electroencephalogram (EEG) is considered an effective tool among neurologists to detect various brain disorders, including epilepsy, owing to its advantages, such as its low cost, simplicity, and availability. In order to reduce the severity of epileptic seizures, it is necessary to design effective techniques to identify the disease at an earlier stage. Since the traditional way of diagnosing epileptic seizures is laborious and time-consuming, automated tools using machine learning (ML) and deep learning (DL) models may be useful. This paper presents an intelligent deep canonical sparse autoencoder-based epileptic seizure detection and classification (DCSAE-ESDC) model using EEG signals. The proposed DCSAE-ESDC technique involves two major processes, namely, feature selection and classification. The DCSAE-ESDC technique designs a novel coyote optimization algorithm (COA)-based feature selection technique for the optimal selection of feature subsets. Moreover, the DCSAE-based classifier is derived for the detection and classification of different kinds of epileptic seizures. Finally, the parameter tuning of the DSCAE model takes place via the krill herd algorithm (KHA). The design of the COA-based feature selection and KHA-based parameter tuning shows the novelty of the work. For examining the enhanced classification performance of the DCSAE-ESDC technique, a detailed experimental analysis was conducted using a benchmark epileptic seizure dataset. The comparative results analysis portrayed the better performance of the DCSAE-ESDC technique over existing techniques, with maximum accuracy of 98.67% and 98.73% under binary and multi-classification, respectively.

## 1. Introduction

Epilepsy is inevitably known to be the most persistent and critical neurological disorder that affects the brain of the human body [1]. It has spread to around fifty million persons of different age groups around the world, with nearly 450,000 persons below the age of 17 out of approximately three million people in the US affected by this disease. Epilepsy is described by its recurring, unprovoked seizures [2]. A seizure is a period of anomalous, synchronous innervation of a population of neurons that may last from a few seconds to minutes. At a molecular level, several pathways were implicated in the apoptosis of premyelinating oligodendrocytes or subplate neurons that exist in perinatal brain growth. A rising concentration of glutamate or free radical reactive species in hypoxic-ischemic encephalopathy, inflammatory cytokines such as TNF-α, IL-1b, IL-6, 12, 15, and 18 from activated microglia and astrocytes, low pH in infections, and free iron secondary to cerebral hemorrhage were broadly stated in both white- and grey-matter injuries as significant triggers for epileptic events [3,4]. An epileptic seizure is an ephemeral instance of complete or partial abnormal, unintended movement of the body that may be integrated with a loss of consciousness [5,6]. Even though epileptic seizures rarely occur in every person, their subsequent effect on social interaction, physical communication, and patient emotion makes the diagnosis and treatment of epileptic seizure of ultimate consequence [7].

Electroencephalograms (EEGs), which have existed for years, are widely employed amongst neurologists to detect different kinds of brain disorders, and especially [8] epilepsy. The wide use of EEG is attributable to possible reasons including its low cost, availability, and effortlessness. EEG is an effective, non-invasive diagnosis method since we can make use of it to accurately denote and capture epileptic signals, i.e., those described by spike-and-wave patterns, sharp waves, or spike difficulties [9,10]. By a visual interpretation of the recorded EEG signal, neurologists can considerably differentiate normal brain activities between seizures (interictal) and epileptic brain activities at the time of seizure (ictal). In the past few years, a large amount of automatic EEG-based epilepsy diagnosis research was conducted [11]. This was inspired by the time-consuming and exhausting nature of the human visual system that is mainly based on medical experts. Moreover, the necessity for effective, objective, and rapid schemes to process large numbers of EEG records has become inevitable to reduce the possibilities of misinterpretation. The availability of this system will considerably improve the living standards of epileptic patients [12].

Epilepsy diagnosis with EEG signal is strenuous and time-consuming since the neurologist or epileptologist needs to minutely screen the EEG signal [13]. In addition, there is a chance of human error, and thus, designing computer-based diagnoses may reduce this problem. Several machine learning (ML) methods were introduced using nonlinear, statistical, and frequency-domain parameters to diagnose epileptic seizures [14]. In traditional ML methods, the classifiers and features selection are obtained using a trial-and-error technique. Likewise, one must have sound knowledge of data mining and signal processing to design an effective method [15]. This method performs well for a small amount of information. Currently, with an increasing amount of data, an ML technique may not work effectively. Therefore, DL methods, that is, an advanced methodology, have been used.

In Almustafa [16], epileptic seizure dataset classification was performed using various classifications. It has been demonstrated that the random forest (RF) method outperforms k-nearest neighbor (KNN), naïve Bayes (NB), logistic regression (LR), decision tree (DT), random tree (RT), J48, and stochastic gradient descent (SGD) classification models. Usman et al. [17] present a method that offers a reliable technique of feature extraction and preprocessing. This method forecasts epileptic seizure with adequate time before the onset of seizure begins and provides a true positive rate. They employed empirical mode decomposition (EMD) for pre-processing and extracted frequency-domain and time features to train a predictive method. The presented method identities the initiation of the preictal state.

In Janghel et al. [18], ANN methods such as back propagation network (BPA), learning vector quantization (LVQ), recurrent neural network (RNN), probabilistic neural network (PNN), and competitive learning (CL) were performed. A primary dataset was used for assessing the presented model. The presented model is able to achieve 97.5% accuracy; the higher accuracy attained confirmed the considerable achievement of the technique. Qureshi et al. [19] aimed at extracting the distinguishing and discriminating features of seizure EEG records to design a model that applies ML and fuzzy-based models for the recognition of epileptic seizures. The presented model categorizes unknown EEG signals into interictal and ictal classes. The method was supported by an empirical assessment on two standard datasets.

Aileni et al. [20] proposed various aspects regarding the detection of epileptic seizures using an EEG signal classification-based supervised learning model with a related learning model that analyzes information. To identify epileptic seizures, a support vector machine (SVM) method was utilized. The SVM method is determined formally by separating hyperplane-based training data (supervised learning), and the model output represents the optimum hyperplane. Abdelhameed and Bayoumi [21] introduce a DL method for seizure detection in pediatric patient-based classification of raw multi-channel EEG signals, i.e., minimally pre-processed signals. The novel technique makes use of the automated feature-learning abilities of a 2D deep convolutional autoencoder (2D-DCAE) connected to neural network (NN)-based classifiers to construct a unified model.

Gao et al. [22] proposed DL-based classifier methods such as epileptic EEG signal classification (EESC). First, this method converts EESC to a power spectrum density energy diagram (PSDED), then exploits deep convolutional neural network (DCNN) and transfer learning (TL) methods for automatically extracting features from the PSDED, and lastly categorizes four classes of epileptic states (seizure, interictal, preictal duration to thirty minutes, and preictal duration to ten minutes). Sarić et al. [23] projected a Field Programmable Gate Array (FPGA)-based solution to classify generalized and focal epileptic seizure types by the use of feed-forward multilayer artificial neural network architecture (MLP ANN). 

The authors in [24] present a cloud-fog integrated smart neurocare model that carries out a temporal investigation of input EEG signals through the use of DL models for epileptic seizure detection. The authors in [25] developed a DL model for epileptic seizure detection on intracranial EEG (iEEG) recordings. This framework performs filtering and segmentation of iEEG signals. At last, the convolutional neural network (CNN) and long short-term memory (LSTM) models are applied for classification. Anter et al. [26] introduced a new epileptic seizure recognition model in the IoT environment, including a hybrid genetic whale optimization algorithm (GWOA) based on naïve Bayes (NB-GWOA) for feature selection, and an adaptive extreme learning machine (ELM) based on a differential evolutionary (DE) algorithm (DEELM) for classification. The authors in [27] developed an epileptic seizure detection and classification model using short-time Fourier transform (STFT)-based denoising, wavelet transform (WT), and a multilayer perceptron neural network (MLPNN) classifier. In [28], the authors initially decomposed the EEG signals through the use of empirical wavelet transform (EWT) with Fourier–Bessel series expansion (FBSE), called FBSE-EWT. In addition, entropy-based features are used, which are then sorted depending upon the p-values attained from the Kruskal–Wallis statistical test to reduce feature space.

Various classification models have been available in the literature to detect and classify epileptic seizures using EEG signals. Despite the benefits of ML and DL models available in the literature, it is required to further improve the classification efficiency. Because of incessant deepening of the model, the number of parameters of DL models also increases rapidly, which leads to model overfitting. On the other hand, several hyperparameters have a considerable influence on the performance of the CNN models. In particular, hyperparameters such as epoch count, batch size, and learning rate selection are essential to attain an effectual outcome. Since the trial-and-error method for hyperparameter tuning is a tedious and erroneous process, metaheuristic algorithms can be applied. Therefore, in this work, we employ a KHA algorithm for the parameter selection of the DSSAE model. 

This paper presents an intelligent deep canonical sparse autoencoder-based epileptic seizure detection and classification (DCSAE-ESDC) model using EEG signals. The proposed DCSAE-ESDC model designs a novel coyote optimization algorithm (COA)-based feature selection model for the optimal selection of feature subsets. In addition, the DCSAE-based classifier is derived for the detection and classification of different kinds of epileptic seizures. Moreover, the parameter tuning of the DSCAE model takes place via the krill herd algorithm (KHA). The intelligent DCSAE-ESDC model consisting of data pre-processing, COA-based feature selection, DCSAE-based classification, and KHA-based parameter tuning is presented. To the best of our knowledge, the ODTLD-CABCC model has been never presented in the literature. The design of the COA-based feature selection and KHA-based parameter tuning shows the novelty of the work. The performance validation of the DCSAE-ESDC model takes place against the benchmark epileptic seizure recognition dataset. 

## 2. The Proposed Model

**Figure 1:**
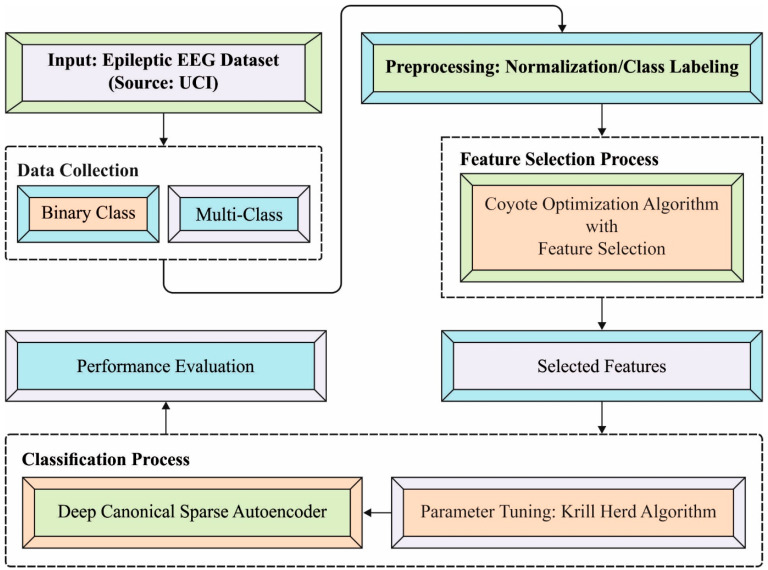
Overall Process of the Proposed Method.

In this study, an effective DCSAE-ESDC model was derived for the identification and classification of epileptic seizures using EEG signals. The proposed DCSAE-ESDC model encompasses several stages of operations, namely, data pre-processing, COA-based feature selection, DCSAE-based classification, and KHA-based parameter tuning. The utilization of a COA-based feature selection approach helps to eliminate the curse of dimensionality problems and enhances the classification results. Figure 1 depicts the overall block diagram of the DCSAE-ESDC model.

### 2.1. Data Pre-Processing

At the initial stage, the EEG signals are pre-processed to improve the signal quality. Initially, minimal–maximal (min-max) method is applied to normalize the dataset. Then, the lower and higher values from the set of information are taken into account. Each piece of information undergoes normalization to this value. The aim is to normalize the maximal value to one and the minimal value to zero, as well as assign other values within [0, 1]. The process of min-max normalization is defined by Equation (1).

(1) $$Min-Max.\;Norm=\frac{x-x_{min}}{x_{max}-x_{min}}\;$$

Finally, the class-labelling process is performed, where the instance in the EEG signal dataset is allotted to proper class labels, with 0, 1, 2, 3, 4 allotted to multi-class and 0, 1 to binary class. 

### 2.2. Process Involved in the COA-Based Feature Selection Technique

After the stage of data pre-processing, the COA is applied to derive an optimal subset of features. The COA is inspired by the dynamic nature of the coyote in an environment and the communication experience of the coyotes [29]. The coyote population can be divided into an equal number of coyotes in every pack. The position of the coyotes is considered an effective solution, and the social state defines the major intention. Initially, the COA starts with the random initiation of coyote locations using Equation (2):

(2) $$X_{i}=lb_{i}+r_{i}\times \left(ub_{i}-lb\right)\;$$

where $ub_{i}$ and $lb_{i}$ represent the maximum and minimum bounds, $r_{i}$ denotes the arbitrary number in [0, 1], and $X_{i}$ represents the location of a coyote. Here, the coyote count for each group is limited to 14, and it verifies the searching capability of the COA. The optimal coyotes can be computed as the optimal ones varied to the environment, followed by the optimum one being considered as the one with the least cost function. In the COA, the coyotes are arranged by contribution to group maintenance and to share the social condition [30]. The social behavior of the group can be determined using Equation (3):

(3) $$Y_{i}^{p,t}=\{\begin{array}{l} C_{\frac{N_{c}+1}{2}}^{p,t},\;N_{c}\;is\;odd\\ \frac{C_{\left(\frac{N_{c}}{2}+1\right),i}^{p,t}}{2},\;Otherwise \end{array}$$

where $N_{c}$ denotes coyote count, $Y_{i}^{p,t}$ signifies the social tendency of the pth pack from the tth time, and C implies the coyote ordered social state. Based on birth and death, the birth of novel coyotes can be determined using Equation (4):

(4) $$B_{i}^{p,t}=\{\begin{array}{l} X_{r1,i}^{p,t},\;r_{j}\geq Pr_{s}+Pr_{a}\;or\;i=i1\\ X_{r2,i}^{p,t},\;r_{i}<Pr_{s}\;or\;i=i2\\ R_{i},\;Otherwise \end{array}$$

where i1 and i2 represent two random dimensions, r1 and r2 suggest the two coyotes randomly selected in the pth pack, $r_{i}$ states the random number generated in [0, 1], $Pr_{a}$ indicates the connection probability, and $Pr_{s}$ shows the scatter probability. $Pr_{a}$ and $Pr_{s}$ can be determined using Equations (5) and (6):

(5) $$Pr_{s}=\frac{1}{D}\;$$

(6) $$Pr_{a}=\frac{1-Pr_{s}}{2}\;$$

In every round, every cth coyote from the pth pack upgrades the social state by the use of Equation (7):

(7) $$X_{c}^{p,t+1}=\{\begin{array}{ll} X_{c}^{p,t}+r1\times \sigma_{1}+r2\times \sigma_{2}, & \;F_{c}^{p,t+1}<F_{c}^{p,t}\\ X_{c}^{p,t},\; & Otherwise \end{array}$$

where $\sigma_{1}$ and $\sigma_{2}$ indicate the alpha and beta pack influence, which is defined using Equations (8) and (9):

(8) $$\sigma_{1}=alpha^{p,t}-X_{r1}^{p,t}\;$$

(9) $$\sigma_{2}=Y^{p,t}-X_{r2}^{p,t}\;$$

where $alpha^{p,t}$ represents the alpha coyote. $F_{c}^{p,t}$. infers the social state cost, which can be determined using Equation (10): 


(10) $$F_{c}^{p,t+1}=f(X_{c}^{p,t})$$

Finally, the optimal coyotes can be selected based on the social criteria as the optimal solution obtained from the problem. 

The FF of the COA aims to determine a solution for attaining a balance between two purposes that are fixed.

(11) $$fitness=\alpha \Delta_{R}\left(D\right)+\beta \frac{\left|Y\right|}{\left|T\right|}$$

where $\Delta_{R}\left(D\right)$ represents the classifier error rate. |Y| indicates the size of subset that the approach selects, and |T| signifies the overall number of features contained from the existing dataset. α indicates the variable ∈[0, 1] compared to the weight of error rate of classifiers, respectively, and β=1−α implies the significance of feande reduction. The classification performance was allowed to reach a significant weight before counting FS. When the evaluation function is able to represent the classification accuracy, the results are void of a solution that is the same as the accuracy; however, it takes the minimal elected feature, which serves as a significant feature to reduce the problem of size.

### 2.3. Process Involved in DCSAE-Based Classification

At this stage, the chosen features are passed into the DCSAE model for the classification process. In the fundamental viewpoint, the AE is an axisymmetric single hidden-layer NN. The AE encoding input sensor data utilizing the hidden layer estimates the minimal error and attains an optimum-feature hidden-layer term. The model of AE derives from the unsupervised computational model of human perceptual learning, which has many functional flaws, i.e., the AE does not learn some practical features with copy as well as input memory to implicit layers, but it could restructure input data with higher precision. The sparse AE inheritance is a concept of AE and establishes the sparse penalty term, increasing constraints to feature learning to a concise term of input data. The CCA is a technique of searching the correlation amongst two kinds of data; however, it could not search a difficult nonlinear connection. To address the restriction of the CCA technique, Andrew et al. [31] developed a DNN for canonical correlation analysis named deep CCA (DCCA). The DCCA has overcome the restriction of CCA that could not identify difficult nonlinear correlations. In DCCA, two DNNs f and g are learned nonlinear representations of all datasets. The DCCA is reached by maximizing the canonical correlation of two DNNs’ outcomes f(X) and g(Y) as:

$$\underset{W_{f},W_{g},U,V}{\max}\frac{1}{N}tr(U^{T}f\left(X\right)g\left(Y{)}^{T}V\right)\;$$

$$s.t.\;U^{T}(\frac{1}{N}f\left(X\right)f\left(X{)}^{T}+rI\right)U=I,$$

$$V^{T}(\frac{1}{N}g\left(Y\right)g\left(Y{)}^{T}+sI\right)V=I,$$

(12) $$u_{i}^{T}f\left(Y\right)g{\left(Y\right)}^{T}v_{j}=0,\;for\;i\neq j.$$

where N implies the entire amount of data, X and Y imply the input matrices of two datasets, I refers to the identity matrix, f and g define the nonlinear representation of two DNNs with parameters $W_{f}$ and $W_{g}$, correspondingly, $U=\left[u_{1,}\ldots ,u_{L}\right]$ and $V=\left[v_{1},\ldots ,v_{L}\right]$ are the CCA directions that present DNN resultants to the top layer with L unit, and $\left(r_{x},r_{y}\right)>0$ are the regularized parameters for instant covariance evaluation [32]. $U^{T}f\left(\cdot \right)$ stands for the nonlinear representations that are utilized in the test. The DCCA attains optimum outcomes once it searches the nonlinear mapping of two kinds of data. Sparse AE gains huge successes from seeking nonlinear compact representation of a single dataset. However, the DCCA cannot obtain an effectual nonlinear dimensional decrease, and SAE cannot search the cross-modal data correlation. DCCA is combined with SAE to obtain the optimum representations of two data kinds and a deep canonically correlated sparse autoencoder (DCSAE) that seeks deep network representations of two datasets to maximize the canonical correlation amongst the two removed top features and minimize the reform errors of sparse AE. The DCSAE is determined as:

(13) $$\begin{array}{c} \underset{W_{f},W_{g},U,V}{\min}-\frac{1}{N}tr(U^{T}f\left(X\right)g\left(Y{)}^{T}V\right)+\frac{\lambda }{N}\|\hat{x^{\left(i\right)}}-x^{\left(i\right)}\|{}^{2}\;\\ +\|\bar{y^{\left(i\right)}}-y^{\left(i\right)}{\|}^{2}+\alpha J_{KL}\left(\rho \|\hat{\rho_{j}}\right)+\beta J_{KL}\left(\sigma \|{\hat{\sigma }}_{k}\right)\; \end{array}$$

where f and g denote the DNNs utilized for extracting nonlinear features to all datasets but encoding all inputs simultaneously. $U=\left[u_{1},\ldots ,u_{L}\right]$ and $V=\left[v_{1},\ldots ,v_{L}\right]$ are the CCA directions that present the DNN outcomes to the top layers with L unit. $\hat{x^{\left(i\right)}}$ and $\hat{y^{\left(i\right)}}$ imply the reform of input $x^{\left(i\right)}$ and input $y^{\left(i\right)}$, correspondingly, and $J_{KL}\left(\cdot \right)$ is determined as $JKL\;\left(\rho \|\hat{\rho_{j}}\right)=\sum_{j=1}^{S}\rho log\frac{\rho }{\hat{\rho_{j}}}+\left(1-\rho \right)log\frac{1-\rho }{1-\hat{\rho_{j}}}$, where $\hat{\rho_{j}}=\frac{1}{N}\sum_{i=1}^{N}h_{j}\left(x^{\left(i\right)}\right)$ signifies the average activation of these hidden units j, similarly to $J_{KL}\left(\sigma \|{\hat{\sigma }}_{k}\right)$.

### 2.4. Parameter Tuning Using KHA

In order to tune the parameters involved in the DCSAE technique, the KHA is applied, thereby boosting the overall classification efficiency. Antarctic krill is the main animal species on Earth. The capability to produce huge swarms is the most important feature of this species. An individual krill will separate from the herd once a predator, namely, seals, whales, and several other species, attack the herd. This attack decreases the density of the KH. The restructuring of the KH following predation affects several parameters. An important objective of the herding performance of krill individuals is to improve krill densities and the realization the food. The KH technique utilizes this multi-objective herd to resolve global optimization issues. To find food (maximum food focus) and density, the dependent attractiveness of the krill is utilized as the objective. Thus, the outcome is that a krill individual changes near optimum solutions once it finds maximum density of herd as well as food. This performance generates a KH near the global minimum of optimized issues [33].

The time-dependent place of individual krill from 2D surfaces is controlled by three subsequent and important activities.

i. An effort made by another krill individual;
ii. Foraging motion;
iii. Physical or random diffusion.

The subsequent Lagrangian technique generalizes to n dimension decision space:

(14) $$\frac{dX_{i}}{dt}=N_{i}+F_{i}+D_{i}\;$$

where $N_{i}$ refers the movement caused by another krill individual; $F_{i}$ implies the foraging movement; and $D_{i}$ stands for the physical diffusion of the *i*th krill individual.

**Figure 2:**
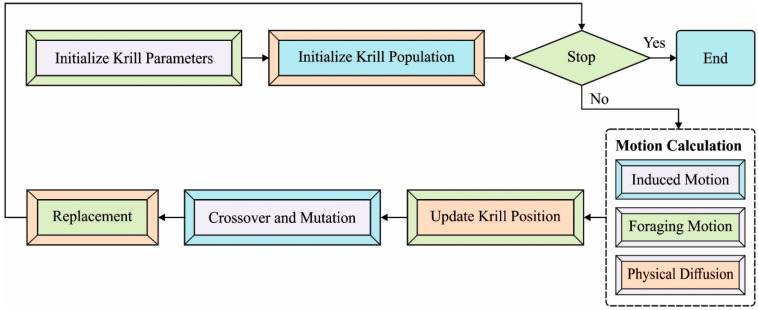
Flowchart of the Krill Herd Algorithm.

The effort of all krill individuals is determined as:

(15) $$N_{i}^{new}=N^{maks}\alpha_{i}+\omega_{n}N_{i}^{old}\;$$

(16) $$\alpha_{i}=\alpha_{i}^{local}+\alpha_{i}^{target}\;$$

where $N^{maks}$ represents the maximal induced speed, and based on the measured values, it could be equal to 0.01 (m/s). $\omega_{n}$ implies the inertia weight of movement induced from the range zero and one. $\alpha_{i}^{local}$ represents the local result provided by neighbors, target indicates the destination result provided by an optimum krill individual, and $N_{i}^{old}$ implies the preceding induced motion [34]. $\omega_{n}$, the inertia weight, is initially equivalent to 0.9 of the optimized. Afterward, it can be linearly reduced to 0.1. Figure 2 demonstrates the flowchart of the KH technique.

The result of neighbors is considered as the attraction or repulsion tendency amongst the individual to local searches. $\alpha_{i}^{target}$, the target outcome offered by optimum krill individuals, is determined as:

(17) $$\alpha_{i}^{target}=C^{best}{\hat{K}}_{i,\;best}{\hat{X}}_{i,best}\;$$

where $C^{best}$ refers to the coefficient of control and is determined as follows:

(18) $$C^{best}=2\left(rand+\frac{I}{I_{maks}}\right)\;$$

where rand stands for the arbitrarily formed number amongst zero and one, I demonstrates the actual iteration number, and $I_{maks}$ defines the maximal number of iterations. 

The KHA approach derives a fitness function to obtain enhanced classification accuracy. It defines a positive integer to characterize the candidate solution performance. In the work, the minimalization of the classification error rate is taken into account as a fitness function. A worse solution achieves an improved error rate, and the optimum solution has a minimal error rate.

(19) $$\begin{array}{c} fitness\left(x_{i}\right)=Classifier\;ErrorRate\left(x_{i}\right)\\ =\frac{number\;of\;misclassified\;samples}{Total\;number\;of\;samples}*100 \end{array}$$

## 3. Results and Discussion

### 3.1. Implementation Data

In this section, the performance validation of the DCSAE-ESDC technique takes place. The DCSAE-ESDC technique was simulated using the Python 3.6.5 tool. The results were tested using a benchmark epileptic seizure recognition dataset from the UCI repository [35]. The dataset involved two versions: a binary class dataset and a multi-class dataset. The details related to the dataset are provided in Table 1. The parameter settings are given as follows: learning rate: 0.01, dropout: 0.5, epoch count: 50, and activation: ReLU. For experimental validation, a 10-fold cross-validation approach was applied.

### 3.2. Result Analysis

Table 2 depicts the FS results of the DCSAE-ESDC technique with other FS models. 

**Figure 3:**
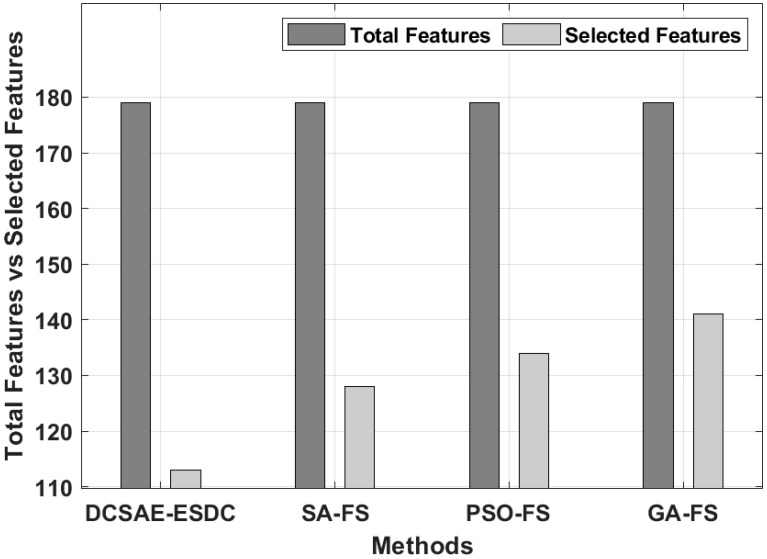
FS analysis of DCSAE-ESDC technique.

Figure 3 provides the number of features chosen by the DCSAE-ESDC technique with other FS models. The figure shows that the GA-FS model elected a higher number of 141 features out of the total 178 features. Likewise, the SA-FS and PSO-FS techniques chose a set of 128 and 134 features, respectively. However, the DCSAE-ESDC technique outperformed the other FS models by choosing the least number of 113 features out of 178 features, which helps to reduce the computation complexity and improve the classification accuracy.

**Figure 4:**
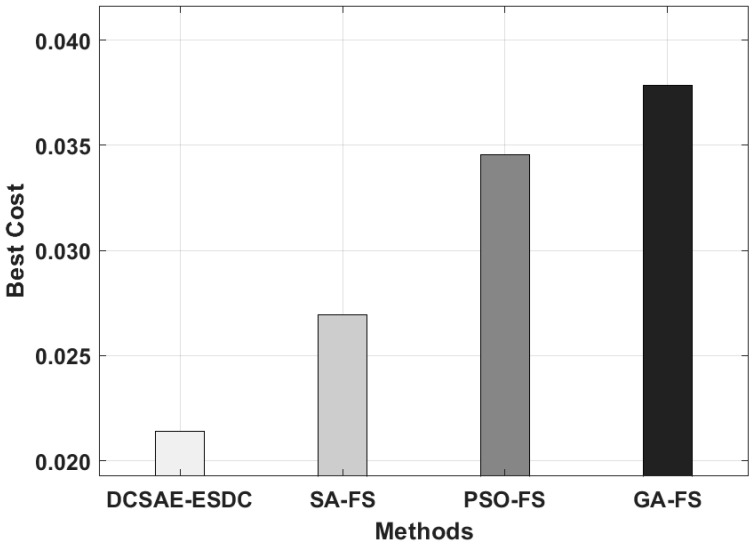
Best cost analysis of DCSAE-ESDC technique.

Next, the best cost analysis of the DCSAE-ESDC technique is shown in Figure 4. The experimental results highlighted that the PSO-FS and GA-FS techniques resulted in poor performance, with the increased BC of 0.0345 and 0.0378, respectively. In line with these, the SA-FS technique tried to obtain a reasonable BC of 0.0269. However, the DCSAE-ESDC technique accomplished a better outcome, with the minimal BC of 0.0214.

Table 3 offers the detailed results analysis of the DCSAE-ESDC technique under the binary class with varying batch sizes (BSs) and epoch counts. The results show that the DCSAE-ESDC technique had the ability to accomplish effective epileptic seizure classification performance in all cases. For instance, with a BS of 32, the DCSAE-ESDC technique reached an average $sens_{y}$, $spec_{y}$, $prec_{n},\;accu_{y}$, $F_{score},$ and MCC of 99.19%, 99.20%, 99.25%, 98.67%, 98.89%, and 99.09%, respectively. Concurrently, with a BS of 64, the DCSAE-ESDC approach attained an average $sens_{y}$, $spec_{y}$, $prec_{n},\;accu_{y}$, $F_{score},$ and MCC of 99%, 99.07%, 99.06%, 98.52%, 98.39%, and 99.05%, correspondingly. Simultaneously, with a BS of 128, the DCSAE-ESDC method obtained an average $sens_{y}$, $spec_{y}$, $prec_{n},\;accu_{y}$, $F_{score}$, and MCC of 98.93%, 99.30%, 99.27%, 98.47%, 98.44%, and 99.12%, correspondingly.

**Figure 5:**
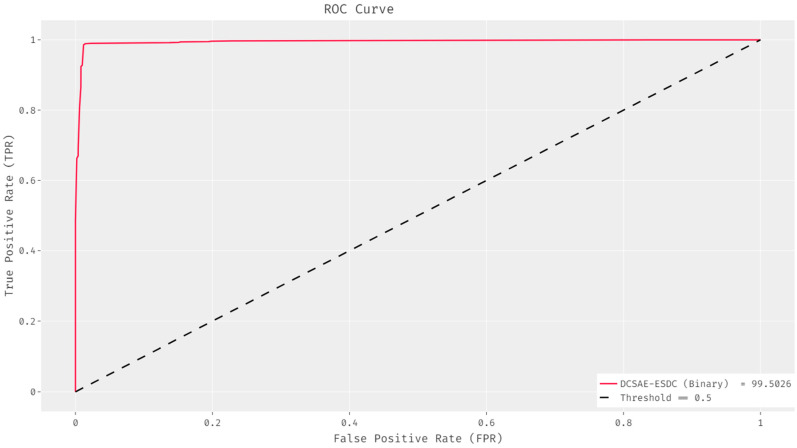
ROC analysis of DCSAE-ESDC technique under the binary class.

An ROC curve (receiver operating characteristic curve) is used to demonstrate the performance of a classification model at all classification thresholds. The ROC analysis of the DCSAE-ESDC technique on the binary classification of epileptic seizures using EEG signals is shown in Figure 5. The figure revealed that the DCSAE-ESDC technique effectively identified the presence of epileptic seizures and attained a maximum ROC of 99.5023.

Table 4 provides the detailed results analysis of the DCSAE-ESDC system under multi-classes with different BSs and epoch counts. The outcomes demonstrated that the DCSAE-ESDC approach had the ability to accomplish efficient epileptic seizure classification performance in all cases. For instance, with a BS of 32, the DCSAE-ESDC algorithm gained an average $sens_{y}$, $spec_{y}$, $prec_{n},\;accu_{y}$, $F_{score}$, and MCC of 99.09%, 99.29%, 99.20%, 98.57%, 98.45%, and 99.12%, correspondingly. With a BS of 64, the DCSAE-ESDC method attained an average $sens_{y}$, $spec_{y}$, $prec_{n},\;accu_{y}$, $F_{score}$, and MCC of 98.96%, 99.26%, 99.19%, 98.73%, 98.44%, and 99.08%, correspondingly. Eventually, with a BS of 128, the DCSAE-ESDC methodology achieved an average $sens_{y}$, $spec_{y}$, $prec_{n},\;accu_{y}$, $F_{score},$ and MCC of 99.02%, 99.11%, 99.25%, 98.65%, 98.82%, and 99.10%, respectively.

**Figure 6:**
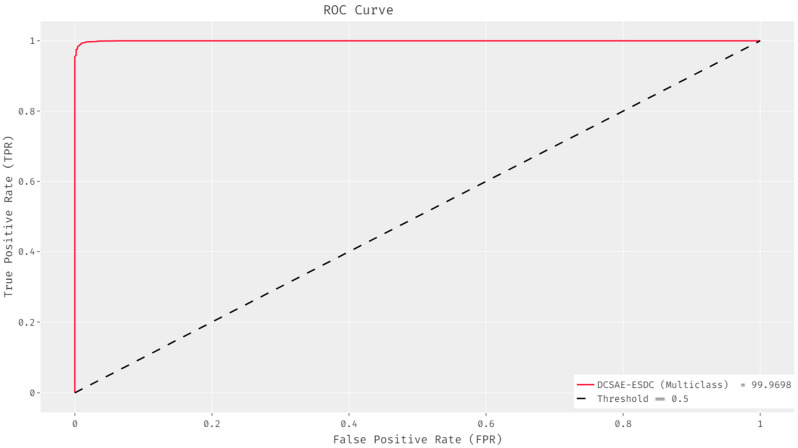
ROC analysis of DCSAE-ESDC technique under multi-class.

The ROC analysis of the DCSAE-ESDC approach on the multi-classification of epileptic seizures utilizing EEG signals is illustrated in Figure 6. The figure states that the DCSAE-ESDC method efficiently identified the presence of epileptic seizures and gained a maximal ROC of 99.5023.

### 3.3. Discussion

**Figure 7:**
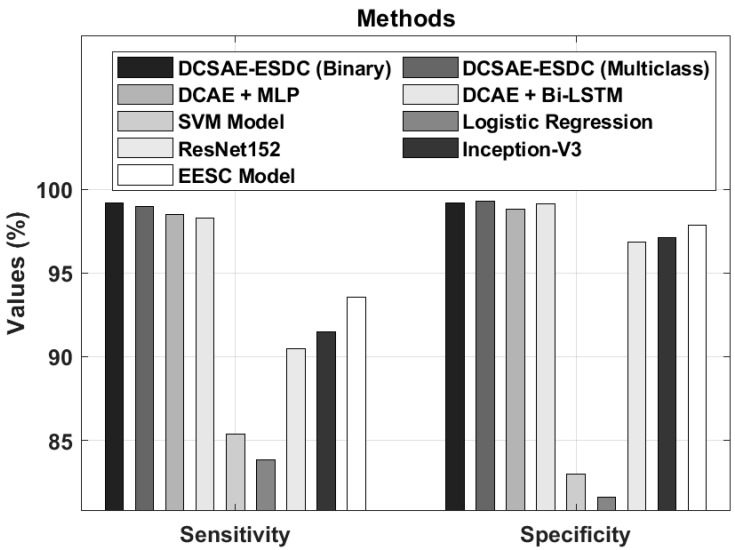
*sens_y_* and *spec_y_* analysis of DCSAE-ESDC technique.

A comparative results analysis of the DCSAE-ESDC technique with recent methods [36] is shown in Table 5. Figure 7 depicts the $sens_{y}$ and $spec_{y}$ analysis of the DCSAE-ESDC approach with existing algorithms. The experimental outcomes exhibited that the SVM and LR methodologies reached minimum classification $sens_{y}$ and $spec_{y}$. In line with this, the ResNet152, Inception-v3, and EESC approaches gained somewhat superior performances. In addition, the DCAE+MLP and DCAE+BiLSTM techniques attained moderately closer $sens_{y}$ and $spec_{y}$ values. At last, the DCSAE-ESDC system resulted in an enhanced classification performance, with the maximal $sens_{y}$ and $spec_{y}$ of 99.19%, 98.96% and 99.20%, 99.26% under binary and multi-class, correspondingly. 

**Figure 8:**
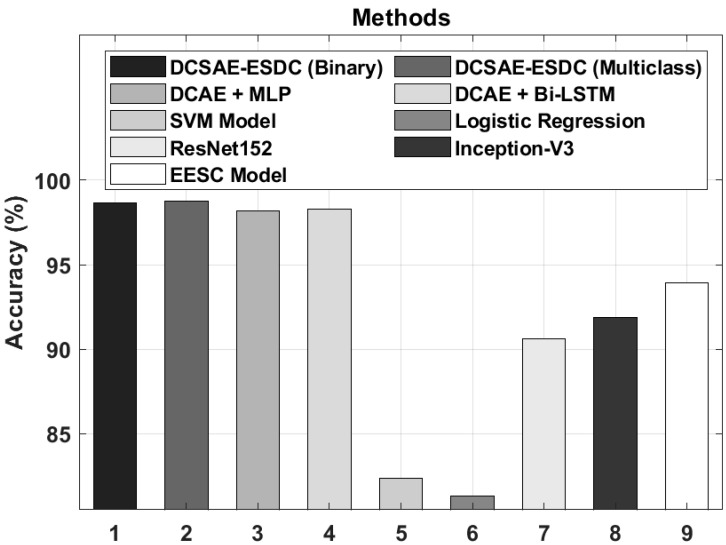
*acc_y_* analysis of DCSAE-ESDC technique with existing approaches.

Figure 8 illustrates the $accu_{y}$ analysis of the DCSAE-ESDC technique with existing techniques. The experimental results demonstrated that the SVM and LR models reached a lower classification $accu_{y}$. The ResNet152, Inception-v3, and EESC techniques obtained slightly improved performances. Moreover, the DCAE + MLP and DCAE + BiLSTM models attained moderately closer $accu_{y}$ values. However, the DCSAE-ESDC technique resulted in an enhanced classification performance, with the maximum $accu_{y}$ of 98.67% and 98.73% under binary and multi-class, respectively. 

**Figure 9:**
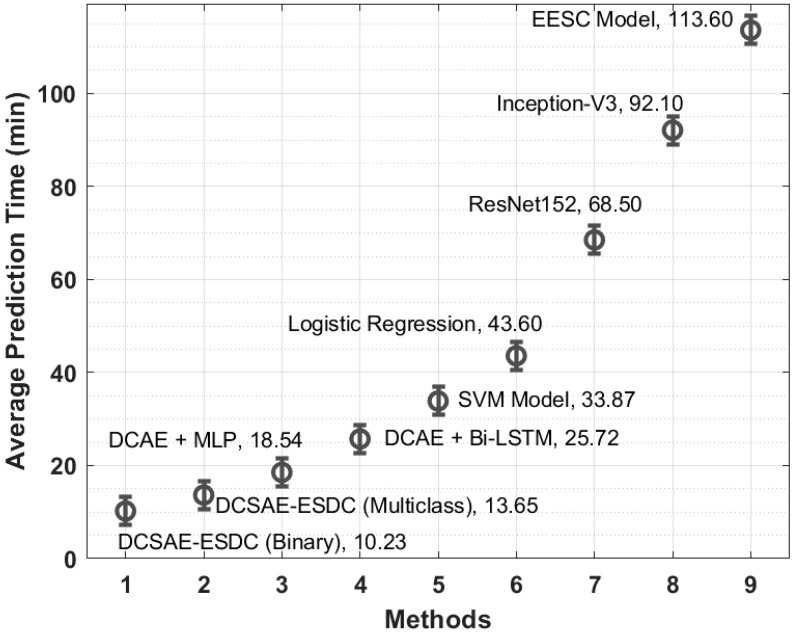
Average prediction time analysis of DCSAE-ESDC technique.

In order to examine the time complexity of the DCSAE-ESDC technique, an average prediction time (APT) analysis of the DCSAE-ESDC technique is shown in Table 6 and Figure 9. The results reported that the EESC, Inception-v3, and ResNet models resulted in lower APTs of 113.60 min, 92.10 min, and 68.50 min, respectively. Along with that, the LR, SVM, and DCAE-BiLSTM models accomplished improved performances with slightly reduced APTs. Though the DCAE + MLP model attained a reasonable APT, the DCSAE-ESDC technique required the least APT of 13.65 min and 10.23 min under multi-class and binary class, respectively.

From the detailed results and discussion, it is ensured that the DCSAE-ESDC technique has been found to be an effective tool to detect and classify epileptic seizures using EEG signals.

## 4. Conclusions

In this study, an effective DCSAE-ESDC technique was derived for the identification and classification of epileptic seizures using EEG signals. The proposed DCSAE-ESDC technique encompasses several stages of operations, namely, data pre-processing, COA-based feature selection, DCSAE-based classification, and KHA-based parameter tuning. The utilization of a COA-based feature selection approach helps to eliminate the curse of dimensionality problems and enhances the classification results. The performance of the DCSAE-ESDC technique was validated against the benchmark epileptic seizure recognition dataset. The experimental outcome stated that the DCSAE-ESDC technique accomplished maximum epileptic seizure classification performance compared to other recent methods, with maximum accuracy of 98.67% and 98.73% under binary and multi-classification, respectively. Therefore, the DCSAE-ESDC technique was found to be useful for epileptic seizure diagnosis in real time. In the future, hybrid DL models with metaheuristics-based hyperparameter optimizers can be designed for improved epileptic seizure detection outcomes.

## Figures and Tables

**Table 1:** Dataset Description.

Class Name	Class Label	No. of Instances
**Binary Class Dataset**		
EEG signals having seizure activity	0	2300
EEG signals not having seizure activity	1	9200
**Multi-Class Dataset**		
EEG signals having seizure activity	0	2300
EEG signals having tumor region	1	2300
EEG signals having healthy brain	2	2300
EEG signals having eyes closed	3	2300
EEG signals having eyes closed	4	2300

**Table 2:** Results Analysis of Applied Feature Selection Methods (Total Features 178).

Methods	Selected Features	Best Cost
DCSAE-ESDC	113	0.0214
SA-FS	128	0.0269
PSO-FS	134	0.0345
GA-FS	141	0.0378

**Table 3:** Results analysis of DCSAE-ESDC technique in terms of distinct measures under the binary class.

Batch Size = 32						
No. of Epochs	Sensitivity (%)	Specificity (%)	Precision (%)	Accuracy (%)	F-Score (%)	MCC (%)
100	99.28	99.27	99.17	98.81	99.04	99.13
200	99.23	98.96	99.15	98.47	98.60	99.11
300	98.92	99.42	99.25	98.37	99.21	99.13
400	99.29	99.20	99.29	98.83	99.03	99.04
500	99.22	99.14	99.39	98.89	98.55	99.04
**Average**	**99.19**	**99.20**	**99.25**	**98.67**	**98.89**	**99.09**
**Batch Size = 64**						
100	98.84	99.10	98.94	98.52	98.09	99.07
200	98.92	99.21	98.95	98.30	98.28	99.01
300	98.90	98.93	99.01	98.89	98.06	99.12
400	99.29	99.11	99.41	98.53	99.00	99.06
500	99.06	99.01	99.00	98.35	98.50	99.00
**Average**	**99.00**	**99.07**	**99.06**	**98.52**	**98.39**	**99.05**
**Batch Size = 128**						
100	99.00	99.44	99.08	98.52	98.07	99.18
200	98.82	99.42	99.40	98.31	98.73	99.03
300	98.87	99.15	99.40	98.39	98.05	99.04
400	98.92	99.42	99.07	98.47	98.17	99.19
500	99.05	99.05	99.39	98.68	99.16	99.16
**Average**	**98.93**	**99.30**	**99.27**	**98.47**	**98.44**	**99.12**

**Table 4:** Results analysis of DCSAE-ESDC technique in terms of distinct measures under multi-class.

Batch Size = 32						
No. of Epochs	Sensitivity (%)	Specificity (%)	Precision (%)	Accuracy (%)	F-Score (%)	MCC (%)
100	98.81	99.32	99.38	98.70	99.23	99.00
200	99.28	99.22	99.33	98.59	98.10	99.14
300	99.21	99.25	99.35	98.56	98.49	99.19
400	99.22	99.40	98.98	98.67	98.41	99.14
500	98.91	99.24	98.96	98.33	98.00	99.14
**Average**	**99.09**	**99.29**	**99.20**	**98.57**	**98.45**	**99.12**
**Batch Size = 64**						
100	99.05	98.97	99.29	98.63	98.08	99.15
200	98.88	99.33	99.14	98.67	98.23	99.00
300	99.02	99.43	99.48	98.57	98.19	99.17
400	98.74	99.33	99.02	98.95	98.77	99.00
500	99.12	99.24	99.02	98.84	98.93	99.07
**Average**	**98.96**	**99.26**	**99.19**	**98.73**	**98.44**	**99.08**
**Batch Size = 128**						
100	98.73	98.97	99.23	98.60	99.30	99.05
200	99.28	98.91	99.21	98.83	99.05	99.11
300	99.28	99.17	99.20	98.86	98.33	99.03
400	99.00	99.40	99.48	98.61	98.56	99.16
500	98.79	99.12	99.15	98.37	98.84	99.14
**Average**	**99.02**	**99.11**	**99.25**	**98.65**	**98.82**	**99.10**

**Table 5:** Comparative analysis of DCSAE-ESDC technique with existing approaches.

Methods	Accuracy (%)	Sensitivity (%)	Specificity (%)
DCSAE-ESDC (Binary)	98.67	99.19	99.20
DCSAE-ESDC (Multiclass)	98.73	98.96	99.26
DCAE + MLP	98.17	98.49	98.83
DCAE + Bi-LSTM	98.26	98.26	99.11
SVM Model	82.39	85.38	83.00
Logistic Regression	81.32	83.85	81.60
ResNet152	90.63	90.45	96.85
Inception-V3	91.89	91.50	97.12
EESC Model	93.92	93.57	97.87

**Table 6:** Average prediction time analysis of DCSAE-ESDC technique with recent methods.

Methods	Average Prediction Time (min)
DCSAE-ESDC (Binary)	10.23
DCSAE-ESDC (Multiclass)	13.65
DCAE + MLP	18.54
DCAE + Bi-LSTM	25.72
SVM Model	33.87
Logistic Regression	43.60
ResNet152	68.50
Inception-V3	92.10
EESC Model	113.60

## Data Availability

The dataset used in this paper is publicly available via the following link: https://archive.ics.uci.edu/ml/datasets/Epileptic+Seizure+Recognition accessed on 16 April 2022.
